# Children’s Beliefs about Pain: An Exploratory Analysis

**DOI:** 10.3390/children8060452

**Published:** 2021-05-27

**Authors:** Lindsay T. Ives, Kate Stein, Alannah M. Rivera-Cancel, Julia K. Nicholas, Kristen Caldwell, Nandini Datta, Christian Mauro, Helen Egger, Eve Puffer, Nancy L. Zucker

**Affiliations:** 1 Department of Psychology and Neuroscience, Duke University, Durham, NC 27708, USA; lindsay.ives@duke.edu (L.T.I.); eve.puffer@duke.edu (E.P.); 2 Department of Psychiatry, University of Oxford, Oxford OX1 2JD, UK; kate.stein@psych.ox.ac.uk; 3 Department of Psychiatry and Behavioral Sciences, Duke University School of Medicine, Durham, NC 27710, USA; alannah.rivera.cancel@duke.edu (A.M.R.-C.); julia.nicholas@duke.edu (J.K.N.); kristen.caldwell@duke.edu (K.C.); christian.mauro@duke.edu (C.M.); 4 Department of Psychiatry and Behavioral Sciences, Stanford University School of Medicine, Stanford, CA 94305, USA; nandinid@stanford.edu; 5 Department of Child and Adolescent Psychiatry, NYU Langone Health, New York, NY 10016, USA; helen.egger@nyulangone.org

**Keywords:** functional abdominal pain, pain, interoception, children, child attitudes, pain thresholds

## Abstract

Functional abdominal pain (FAP) is one of the most common childhood medical complaints, associated with significant distress and impairment. Little is known about how children understand their pain. Do they attribute it to personal weakness? Do they perceive pain as having global impact, affecting a variety of activities? How do they cope with pain? We explored the pain beliefs of 5- to 9-year-old children with FAP using a novel Teddy Bear Interview task in which children answered questions about a Teddy bear’s pain. Responses were analyzed quantitatively and qualitatively. Results indicate that the majority of young children with FAP are optimistic about pain outcomes. Children generated many types of coping strategies for Teddy’s pain and adjusted their calibration of Teddy’s pain tolerance dependent on the activity being performed. Early warning signs also emerged: a subset of children were pessimistic about Teddy’s pain, and several children identified coping strategies that, while developmentally appropriate, could lead to excessive help seeking if not intervened upon (e.g., physician consultation and shot). The Teddy Bear Interview allows children to externalize their pain, making it a useful tool to access cognitive pain constructs in younger children. Thus, these findings highlight the importance of early intervention for childhood FAP.

## 1. Introduction

Functional abdominal pain (FAP) is one of the most common physical complaints of childhood [1]. FAP is one of the class of disorders of gut–brain interaction: GI disorders resulting from the interaction of gut physiology (e.g., disturbances in motility, altered sensory experience, immune dysfunction) with central nervous system processing. According to the Rome IV Criteria, FAP can be defined as when abdominal pain occurs 4 or more times a month, for at least 2 months, in either an episodic or continuous fashion, and cannot be ascribed to another medical condition [2]. A meta-analysis pooling prevalence rates across 58 studies, summarizing data from 196,472 children aged 4 to 18 years, found that 13.5% of youth (1.6% to 41.2% across studies) suffer from FAP disorders worldwide [1]. Studies using Rome III criteria [3], the diagnostic criteria for functional gastrointestinal disorders used in this analysis, had higher prevalence estimates at 16.4%. Prevalence rates were similar when data were collapsed for children below and above 12 years of age, suggesting that FAP is common across early development. Based on the data summaries in this meta-analysis, approximately 1 in 10 children experience FAP.

The presence of FAP in childhood increases vulnerability for several adverse outcomes in adulthood including chronic pain and persisting mental health conditions [4,5,6,7]. Individual differences may exacerbate these vulnerabilities: children with FAP who also have a negative attributional style (i.e., those who attribute adverse events to personal, global, and stable factors rather than external, local and unstable factors; explained further below) [8] are particularly susceptible to depression or anxiety disorders at age 18 [6]. Given the significant number of children diagnosed with FAP and the prognostic significance of this condition, early intervention is essential to help children (and their parents) manage pain immediately, with implications for preventing more chronic disorders [9]. Additionally, understanding individuals’ beliefs about their pain across development should inform timing and targets of intervention.

The majority of interventions that have been developed for FAP are not specifically targeted to younger children [10,11,12]. There have been important advances in pain treatment for children and adolescents [13,14]. Current cognitive behavioral treatments cater to older children, and among the tools provided, there is an emphasis on reducing pain catastrophizing in parents and providing the children with distraction and relaxation techniques [15]. These treatments are generally effective in reducing the child’s pain; however, their associated depression and anxiety does not often respond to treatment [15]. The strategies applied may not be optimal for young children: the focus on distraction from the pain has led to some concern that the treatment does not modify the amount of pain experienced by the child, but only the amount of pain reported to parents [15]. Furthermore, there is evidence that a significant proportion of children fail to respond to the most effective cognitive-behavioral therapy (CBT) interventions for FAP. Across randomized controlled trials of CBT interventions in children, there is significant variation; approximately 9.4–68.2% of individuals fail to demonstrate an optimal treatment response [16,17,18,19,20].

### 1.1. Explanatory Styles

Individual difference factors, such as beliefs about pain stability (i.e., whether the pain will persist indefinitely), may influence response to treatment in FAP. The importance of explanatory (also referred to as attributional) style in internalizing disorders, such as depression and anxiety, is well established, and there is now growing interest in how one’s explanatory style is associated with prevalence rates of chronic pain in childhood [6]. A negative explanatory style is a person’s tendency to attribute adversity to global, stable or personal factors. Such explanatory styles may impact the pain experience [6]. For example, does a child believe that their abdominal pain causes problems in all areas of their life (global); do they attribute their abdominal pain to the fact that they are weaker than others (personal); and do they believe that they are always going to be sick (stable)?

Targeting younger children may be a particularly advantageous strategy in managing FAP. Children’s explanatory styles, as well as thinking about illness-related concepts such as pain, develop over time [21,22]. Early beliefs about pain may have less impact and may be more amenable to intervention than more long-standing explanatory styles in adolescence and adulthood. Furthermore, children are just learning to decode the meaning of bodily sensations. One developmental task of young children is learning to contextualize divergent body sensations, interpret the meaning of these sensations, and act on that interpretation in an adaptive way [23,24]. For example, a child may notice a fluttering in her gut in her kindergarten class as she practices writing, label that she is nervous, and exchange a smile with a friend. Thus, the strategies for managing pain in older children may not be suited for younger children, who may miss this important developmental skill if just advised to distract from pain. As we develop age-specific interventions, it is important to understand the nature of children’s experience and understanding of pain.

Previous studies of children’s beliefs about pain have had important prognostic implications. One study found that the more severe 8- to 17-year-old children perceived their pain to be, the more likely they were to be taken to a physician by their caregiver during a given three-month period [25]. Another study showed that 8- to 18-year-old children and adolescents with FAP have different coping strategy profiles, including avoidant, dependent, self-reliant, engaged, infrequent, and inconsistent [26], and that their styles of coping were associated with different levels of perceived coping efficacy (e.g., avoidant and dependent copers had lower levels of efficacy; self-reliant and engaged copers had higher levels of efficacy). Additionally, child catastrophizing of pain has been shown to be related to higher levels of functional disability, depression, and anxiety in a sample of children aged 7 to 17 years [27]. These studies highlight the wide-ranging relations that children’s beliefs about pain have with various areas of life, including health care utilization, pain management, impairment, and psychopathology.

Children’s pain beliefs have previously been measured using the Pain Response Inventory, Pediatric Pain Beliefs Questionnaire, and the Pediatric Version of the Survey of Pain Attitudes [28,29,30]. These measures rely on children’s self-report using a questionnaire, and none of them have been normed on children younger than 8 years old. Additionally, while the Pain Beliefs Questionnaire has a parent component, the Emotion-Focused Coping Efficacy subscale of the Pain Beliefs Questionnaire was found to have weak correspondence between child and parent report, conceivably because it measured internal, less observable behaviors. Relatedly, meta-analysis of 341 studies found low correspondence between multiple informants, including parent–child pairs, on internalizing concerns [31]. While each informant provides different information that should be taken into consideration, it is important to develop self-report methods for young children regarding their experiences of their pain to ensure their own perspectives are better understood. The current study uses a novel interview format that integrates a concrete task to assist younger children to access their beliefs about and coping strategies for pain.

### 1.2. Current Study

The current study builds on prior work by examining pain beliefs in children. One method to assess a young child’s beliefs about abdominal discomfort is to externalize the pain onto a familiar yet distinct object, such as a Teddy bear. The ‘Teddy Bear Hospital’ is a well-established worldwide project, which aims to reduce young children’s fears of medical procedures and to enhance their health knowledge [32]. Children who visit the Teddy Bear Hospital have been shown to have significantly better knowledge concerning their body, health, and disease [33], and medical play employed by the Teddy Bear Hospital can be helpful in reducing a child’s anxiety related to the health care encounter [32].

We utilized the concept of the Teddy Bear Hospital tool in a novel way to explore young children’s beliefs about pain as part of an intake interview for a clinical trial to address FAP in children. We assessed their beliefs regarding pain-related distress and impairment at school, home- and fun-related activities (global); ideas about weakness (personal); pain stability (stable); and their coping strategies (Appendix A). We sought to explore not only the children’s pain beliefs, but also to test the feasibility of the Teddy Bear Interview as a method of measuring these beliefs.

### 1.3. Objectives

Given that the Teddy Bear Interview is a novel task and that previous measures of children’s pain have not been validated to children under the age of 8 years, the analyses in this paper are exploratory in nature. Consistent with the objectives of an exploratory investigation [34], we refrained from hypothesis testing and had the following objectives. First, we sought to characterize what proportion of children endorsed more positive or negative explanatory styles related to pain, as well as age differences in these beliefs. Secondly, we explored perceived pain tolerance in various contexts (e.g., school, exercise, play, eating). Third, we investigated relations among children’s pain frequency, intensity, and beliefs. Finally, qualitative analyses were used to explore themes related to children’s coping methods, functional impairment, and pain experience.

## 2. Materials and Methods

### 2.1. Overview

This interview was part of a randomized clinical trial probing two intervention strategies for FAP in young children 5 to 10 years of age at the time of recruitment. The interview was added to the trial in the latter phases of the project and thus represents a subsample of total participants before undergoing intervention. We briefly describe this trial and the development of this interview regarding child beliefs related to pain experience. For a description of the experimental treatment, see Zucker et al. [24].

### 2.2. Recruitment

Research staff recruited on site at pediatric primary care practices within an academic health system in the Southeastern United States. Research staff integrated with the clinic patient flow, having nurses approach caregivers to assess interest in learning about a treatment study for abdominal pain. Interested parents were then screened by study staff assessing the frequency and severity of their child’s episodes of abdominal pain based on a short screener derived from the Questionnaire on Pediatric Gastrointestinal Symptoms based on Rome III Criteria [35]. Families were deemed eligible if their child either had 2 or more stomach aches associated with impairment or the child had 8 or more stomach aches with or without any associated impairment over a two-month period. In order to be considered potentially eligible, all children were between the ages of 5 and 10 years old and had not been diagnosed with intellectual disability (IQ < 70). These criteria were reviewed through children’s medical charts. Study information was also submitted to electronic newsletters of the local school system, and primary care providers throughout the health system were informed of this study for referrals.

### 2.3. Assessments

#### 2.3.1. Teddy Bear Pain Interview

**Figure 1:**
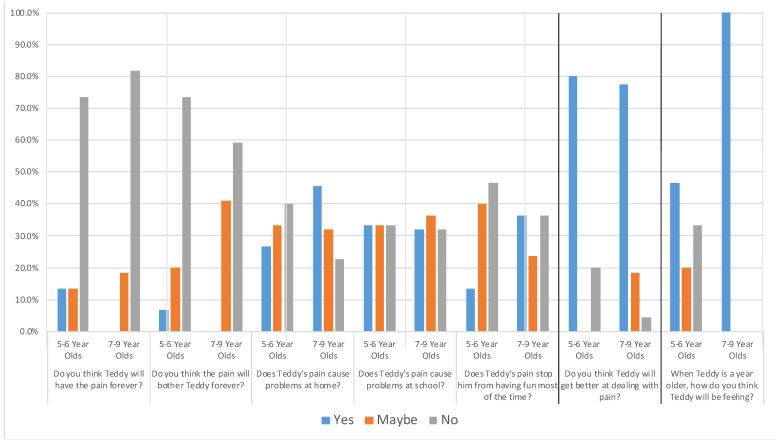
Frequency of Children’s Responses to Teddy Bear Abdominal Pain Interview by Age. Note. For all but one question, bars reflect the percentages of children who responded yes/maybe/no to each question asked by the interviewer. For the question “When Teddy is a year older, how do you think Teddy will be feeling?”, blue bars reflected “better,” orange “the same,” and gray “worse.” The heavy lines are used to highlight that the question second from the right was worded in the opposite direction relative to the questions to the left: lower scores are more positive, and the question furthest to the right had different responses.

Children were interviewed about pain beliefs indirectly: by posing as a “doctor” and answering questions about the experience of their “patient,” a stuffed Teddy bear. Trained research coordinators and clinical psychology graduate students administered the interview. Parents were present in an adjoining room, visible to the children but not participating in the interview. Children were told that they were going to be the doctor and were given the option to wear a doctor’s outfit and stethoscope. They were told that “Teddy” was their patient and experienced pain in the same manner as the child (“has pain just like you have”). Through a structured “Teddy Bear Interview” children provided information about their beliefs about Teddy’s pain. This framework allowed children a more concrete way to conceptualize their beliefs and explanatory styles related to their own pain. Question formats included open-ended, multiple choice (3 options), and indications of pain severity on a pain thermometer (Appendix A). Throughout the interview, questions relating to the following domains were asked: general pain information (e.g., “Can you think of ways to make Teddy’s pain better?”), beliefs about personal factors related to pain (e.g., using a “Teddy bear Likert scale” children were asked to “Point to the Teddy that shows how strong or weak you think Teddy is compared to other Teddies.”), beliefs about the stability of the pain (e.g., “Do you think Teddy will have the pain forever?”), and beliefs about the global impact of their pain (e.g., “Does Teddy’s pain stop him from having fun most of the time?”). Most 3-option multiple choice questions included the responses “yes,” “maybe,” and “no,” except for the question “When Teddy is a year older, how do you think Teddy will be feeling?” which included “better,” “the same,” and “worse.” The interview took approximately 10 to 15 min to complete with some variability depending on a child’s level of cooperation and engagement. See Figure 1 for an excerpt of interview questions and Appendix A for the complete Teddy Bear Interview.

#### 2.3.2. Tolerance of Pain in Diverse Contexts

Using a pain thermometer that used color (going from white to increasing intensities of red), number (0 to 12), and verbal prompts (no pain to the worst pain imaginable), children were asked to indicate how much pain Teddy could experience and still be able to perform certain activities [36].

#### 2.3.3. Preschool Age Psychiatric Assessment (PAPA) Interview

The PAPA, based on the Child and Adolescent Psychiatric Assessment, is a parent-report semi-structured diagnostic interview administered by trained interviewers, designed to assess the feelings and behaviors pertinent to young children [37]. The PAPA diagnoses psychiatric disorders in young children with symptom items coded such that each symptom, if coded as present, has exceeded a diagnostic threshold whereas diagnoses can be determined by algorithm in correspondence with the existing diagnostic classification system for psychiatric disorders [38]. Evidence of its construct and predictive validity has been demonstrated [38,39]. The sections on anxiety disorders, affective disorders, and psychosomatic disorders were administered. The interview covers a time interval of three months. For this study, we report on the child’s frequency of abdominal pain episodes during the three months prior to treatment initiation.

#### 2.3.4. Pain Diaries

Parents were given diaries to record data related to their child’s pain experiences during the seven days before beginning treatment. They completed responses in the diary three times a day (beginning of day, before dinner, and end of day). Abdominal pain intensity was measured using the same 13-point thermometer described above, using both parent and child report at the beginning of the day and before dinner. At the end of the day, parents rated the highest level of abdominal pain that the child experienced throughout the entire day. Pain diaries were completed before the Teddy Bear Interview and PAPA.

#### 2.3.5. Anxiety and Depressive Symptoms

Parent proxy report measures of anxiety and depressive symptoms were taken from the Patient Reported Outcomes Measurement Information System (PROMIS), a collection of self-report and parent proxy report measures designed to assess physical, mental, and social health in adults and children. This system of measurement provides reliability and validity at both the item and scale levels to allow for flexible assessment of variables of interest. As part of the baseline questionnaire battery, parents completed the 8-item PROMIS Parent Proxy Short Form v1.0–Anxiety 8a and the 6-item PROMIS Parent Proxy Short Form v1.0–Depressive Symptoms 6a [40]. Items are answered on 5-point scale ranging from “Never” to “Almost Always” with higher scores indicating greater severity (range: 0 to 32, anxiety; 0 to 24, depression) and per scoring instructions, raw scores are converted to T-scores (see Table 1). In a sample of 82 children with chronic pain, the pediatric PROMIS anxiety and depressive symptoms scales demonstrated convergent validity with validated measures of anxiety [41] and depressive symptoms [42,43]. Scores on these parent proxy measures of anxiety and depressive symptoms were correlated with scores on the corresponding pediatric self-report measures in a study of 1548 parent–child pairs [44].

### 2.4. Data Analysis

#### 2.4.1. Quantitative

Analyses were conducted using IBM^®^ SPSS^®^ Statistics Version 26. A one-sample t-test was conducted to determine whether the mean age of children who completed the Teddy interview differed significantly from the mean age of all children who participated in the randomized clinical trial. A chi-squared test was conducted to determine whether the distribution of race, ethnicity, and sex of children who completed the Teddy interview differed from these distributions for all children who participated in the randomized clinical trial. Means, frequencies, and percentages of children’s beliefs about Teddy’s pain before treatment were calculated. Spearman’s correlations were run to measure the relation between children’s ages and their beliefs. Paired-samples t-tests were used to compare the degree of pain that the child thought Teddy could tolerate and still engage in activities relative to the pain Teddy could tolerate and attend school. Spearman’s correlations were run comparing the frequency of tummy pain during the three months before beginning treatment, as well as the intensity of tummy pain during the seven days prior to treatment, to the children’s beliefs at baseline. This sample did not contain missing data.

#### 2.4.2. Qualitative

Children’s responses to interview questions regarding their beliefs about pain were analyzed qualitatively using NVivo^®^12. Two independent coders read and identified themes in the children’s interview responses and subsequently created a codebook to define the boundaries of identified code [45]. Children’s interview responses were coded by the two coders working independently using this codebook. After the first round of coding, the coders met to revise the codebook. Themes identified during the coding process that were not included in the initial codebook were added and defined. Several code definitions were revised to create more clearly distinguished categories. All interview responses were then re-coded by the two coders working independently using the revised codebook. Following re-coding, a coding comparison query was conducted in NVivo 12^®^. The two coders met to reconcile discrepancies in coding until 100% agreement was reached.

## 3. Results

### 3.1. Demographic Information of Teddy Bear Interview Sample

Table 2 presents the demographic information from the entire sample that participated in the clinical trial for FAP as well as the subset of children that participated in the pain interview. Participants were children (15 boys and 23 girls) aged 5 to 9 years (M = 7.5, SD = 1.4). Thirty-two participants identified as white, five as Black, one as multiracial, and one as Hispanic. There were no significant differences in sex, age, race, and ethnicity between the sample used for the Teddy Bear Interview and the larger participant cohort. Results of the t- and chi-squared tests showed that children participating in the interview did not statistically differ in age or sex, gender, or ethnicity from the entire sample, *p* > 0.2.

Anxiety raw scores on the PROMIS measure ranged from 0 to 17. Corresponding standardized T-scores ranged from within normal limits (T = 34, SE = 6) to severe (T = 65, SE = 3). Depressive symptom raw scores on the PROMIS measure ranged from 0 to 14. Corresponding standardized T-scores ranged from within normal limits (T = 36, SE = 6) to severe (T = 67, SE = 3). See Table 1 for means, percentages, and frequencies of scores.

### 3.2. Baseline Beliefs by Age

Before undergoing treatment, the majority of children were optimistic about the potential for Teddy’s pain to improve. They answered questions about Teddy’s pain on a 3-point scale that included the responses “yes,” “maybe,” and “no.” Seventy-three percent of 5- to 6-year-olds and 82% of 7- to 9-year-olds believed that Teddy would not have the pain forever. Seventy-three percent of 5- to 6-year-olds and 59% of 7- to 9-year-olds believed that the pain would not bother Teddy forever. Eighty percent of 5- to 6-year-olds and 77% of 7- to 9-year-olds reported that Teddy would get better at dealing with the pain. Children’s responses were mixed when asked about whether Teddy’s tummy pain caused problems at home or school or stopped him from having fun most of the time, with approximately one-third of children in each age group indicating “yes,” “maybe,” and “no.” All 7- to 9-year-olds predicted that Teddy would be feeling better when he was a year older, while approximately one-third of 5- to 6-year-olds indicated that he would that he would feel “better,” “the same,” and “worse.” Frequencies of children’s responses at baseline are presented in Figure 1.

Spearman’s correlations were run to determine the relationship between children’s ages and their beliefs about their pain. There was a significant positive association between age and beliefs about how Teddy would be feeling when he is a year older, (*rs*(36) = −0.64, *p* < 0.001): the older the children were, the more likely they were to believe that Teddy would be feeling better. There was a significant positive association between age and the threshold of pain that Teddy could experience and still engage in various activities, (*rs*(36) = 0.35, *p* = 0.03): the older the children were, the more pain they believed Teddy could have pain and still engage in activities.

### 3.3. Tolerance of Pain in Diverse Contexts

A paired-samples t-test was used to compare the degree of pain that the child thought Teddy could tolerate and still engage in activities. Pain thresholds for other activities were compared to the level of pain that the child indicated Teddy could tolerate and still go to the school. Relative to school (*M* = 6.1, SD = 3.0), children reported Teddy being able to tolerate more pain if they had to watch television (*M* = 8.3, SD = 3.8), *t*(37) = −2.8, *p* = 0.008, *d* = 0.62, or eat a healthy food (*M* = 8.2, SD = 4.2), *t*(37) = −3.1, *p* = 0.004, *d* = 0.59. For the remaining situations, children reported that Teddy could tolerate less pain, though findings were not significant (reading a book, *d* = 0.33; playing with a friend, *d* = 0.06; eating a favorite food, *d* = 0.17; playing a favorite sport, *d* = 0.38).

**Figure 2:**
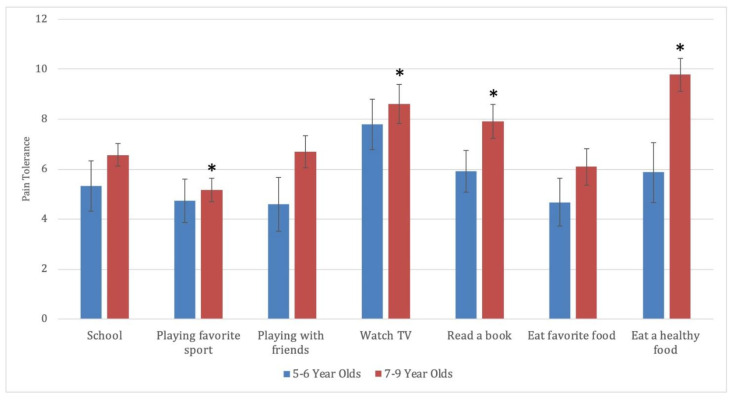
Toleration of Pain in Diverse Situations. Note. Children were asked how much pain Teddy could have and still engage in various categories of activities. Children indicated these pain thresholds on a pain thermometer numbered from 0 to 12 with higher numbers (as also indicated by verbal descriptions and colors) as indicating greater pain. All categories were compared to the level the children indicated Teddy could tolerate and still attend school. Error bars represent the standard errors of the mean. * = significance level is *p* < 0.05.

An additional exploratory analysis of differences in pain thresholds for was run considering age groups separately. Results remained significant for 7- to 9-year-olds with two additional activities reaching significance. Relative to school, (*M* = 6.6, SD = 2.1), 7- to 9-year-olds reported Teddy being able to tolerate more pain and watch television (*M* = 8.6, SD = 3.8), *t*(22) = −2.6, *p* = 0.018, *d* = 0.53; read a book (*M* = 7.9, SD = 3.2), *t*(22) = −2.5, *p* = 0.019, *d* = 0.53; or eat a healthy food (*M* = 9.8, SD = 3.1), *t*(22) = −5.9, *p* < 0.001, *d* = 1.23. They also indicated Teddy could tolerate less pain to play a favorite sport (*M* = 5.2, SD = 2.2), than go to school, *t*(22) = 2.4, *p* = 0.024, *d* = 0.50. Results are presented in Figure 2.

### 3.4. Associations with Pain

Spearman’s correlations were run comparing the intensity and frequency of children’s abdominal pain to their pain beliefs. There was a significant negative association between average parent-rated pain intensity in the morning during the seven days before treatment and children’s beliefs about whether tummy pain caused problems at school, (*rs*(36) = −0.323, *p* = 0.048). The higher a parent rated their child’s pain in the morning, the more likely their child was to believe that Teddy’s pain caused problems at school. There was also a significant positive association between average parent-rated pain intensity for the entire day during the seven days before treatment and children’s beliefs about the stability of the pain, (*rs*(36) = 0.360, *p* = 0.026). If children had higher peak levels of parent-rated pain intensity over the course of the day, they were more likely to believe that Teddy would not have the pain forever. None of the correlations between frequency of pain episodes and children’s pain beliefs were found to be statistically significant, (*rs*(36) = −0.229–0.161, *ps* > 0.05). See Table 3 for an overview of all the correlations.

### 3.5. Qualitative Results

#### 3.5.1. Pain Reduction Strategies

**Figure 3:**
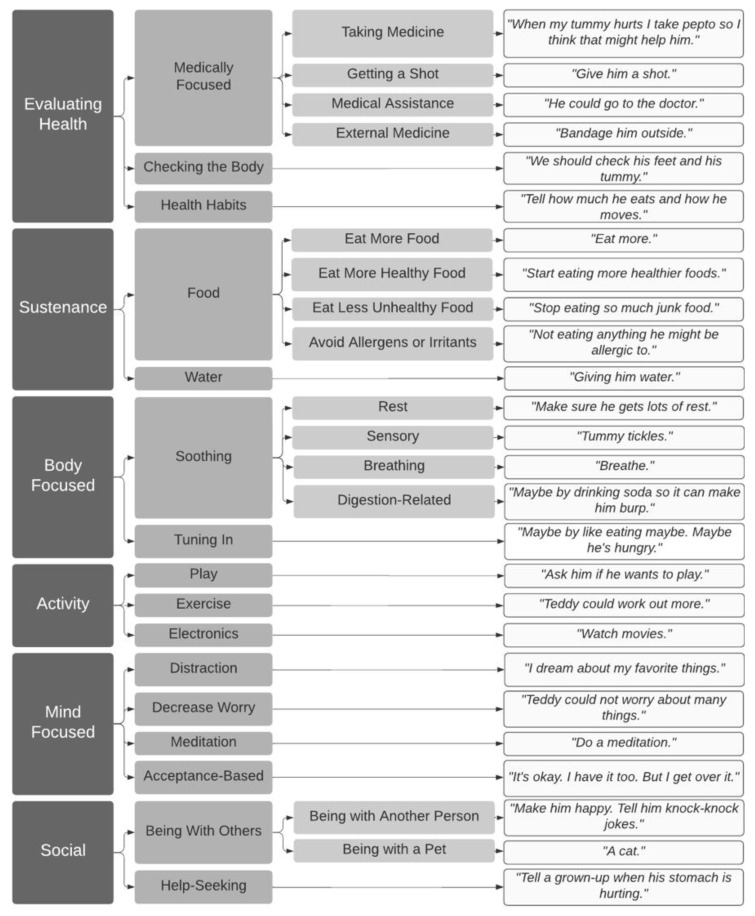
Children’s Ideas Regarding Pain Reduction Strategies. Note. Children’s open-ended responses to the question “Can you think of ways to make Teddy’s pain better?” were coded. Moving from left to right, broader categories (e.g., body-focused strategies) are divided into more specific sub-categories, with sample quotes from each sub-category furthest on the right.

Eighty percent of children (73% of 5- and 6-year-olds, *n* = 11; 87% of 7- to 9-year-olds, *n* = 20) proposed at least one strategy to reduce Teddy’s pain. The remaining 20% of children (*n* = 7) reported that they did not know or that they could not think of any ways to make Teddy’s pain better. The strategy classification scheme, as well as response examples for each category, are detailed in Figure 3.

Children proposed a variety of strategies to alleviate Teddy’s pain in response to the question “Can you think of ways to make Teddy’s pain better?”. Fifty-eight percent of children (*n* = 22) suggested strategies related to evaluating Teddy’s health. Some health evaluation strategies involved checking Teddy’s body parts to identify the cause of pain (e.g., “We should check his feet and his tummy;” 11% of children, *n* = 4) or checking in about Teddy’s health habits (e.g., “Tell how much he eats and how he moves;” 3% of children, *n* = 1). Fifty percent of children (*n* = 19) proposed strategies that involved seeking medical care, including taking medicine at home (42%, *n* = 16), getting a shot (8%, *n* = 3), seeking outside medical assistance (5%, *n* = 2), or using external medical treatments (e.g., bandages; 3%, *n* = 1). Eighteen percent of children (*n* = 7) only proposed these types of strategies (23% of those that proposed a strategy at all).

Food and water were also common themes in children’s pain reduction strategies. Twenty-nine percent of children (*n* = 11) suggested strategies related to nutrition and hydration, such as eating more healthy food (13%, *n* = 5), drinking water (11%, *n* = 4), eating less unhealthy food (8%, *n* = 3), avoiding allergens and irritants (5%, *n* = 2), and eating a greater quantity of food (3%, *n* = 1).

Twenty-nine percent of children (*n* = 11) suggested an array of body-focused pain reduction strategies. Some of these strategies were soothing, including rest (16%, *n* = 6) and sensory approaches (e.g., giving Teddy a pacifier or tummy tickles; 8%, *n* = 3). Other body-focused strategies related to tuning in to one’s body (e.g., doing breathing exercises, trying to track or understand the pain, going to the bathroom; 11%, *n* = 4).

Eleven percent of children (*n* = 4) suggested engaging Teddy in activities such as play (5%, *n* = 2), exercise (3%, *n* = 1), and watching movies (3%, *n* = 1).

Additionally, 8% of children (*n* = 3) suggested mind-focused tactics, including distraction (e.g., “Count backwards from 100;” 5%, *n* = 2), decreasing Teddy’s worry (3%, *n* = 1), taking an acceptance-based approach (e.g., “It’s okay. I have it too. But I get over it;” 3%, *n* = 1), and doing a meditation (3%, *n* = 1).

Finally, 8% of children (*n* = 3) suggested social strategies for helping Teddy’s pain go away, including seeking help from others (3%, *n* = 1) or simply being with other people or pets (5%, *n* = 2).

#### 3.5.2. Pain Interference

Seventy-six percent of children (*n* = 29) identified another activity, in addition to the ones previously asked about, that Teddy’s pain got in the way of. They identified many daily functions, including health behaviors such as diet (11%, *n* = 4), exercising (11%, *n* = 4), and sleeping (3%, *n* = 1); school-related activities (8%, *n* = 3); recreational activities such as arts and crafts (3%, *n* = 1), using electronics (3%, *n* = 1), playing (11%, *n* = 4), performing (3%, *n* = 1), reading (3%, *n* = 1), sports (16%, *n* = 6), going for outings (3%, *n* = 1), and traveling (3%, *n* = 1); and social interaction with family (3%, *n* = 1).

## 4. Discussion

This study employed a Teddy Bear Interview as a means to explore the content of young children’s thoughts about pain. In general, findings suggest that children were optimistic about Teddy’s prognosis and were able to generate creative coping strategies for pain. A subset of children endorsed pessimistic beliefs about pain. Some correspondence between pain intensity and the child’s beliefs about the Teddy bear’s pain, as well as age-related differences in pain beliefs, were found. Children were also able to calibrate different levels of pain Teddy could tolerate while engaging in various activities. Overall, findings in this study indicate the novel Teddy Bear Interview paradigm may allow children to externalize and explore their beliefs about pain. Additionally, preliminary findings highlight the potential benefits of early intervention for children with FAP.

Results from this study suggest that younger children with FAP may be more optimistic than older children about the stability of pain. Whereas in this study, the majority of children indicated that Teddy’s pain, pain distress, and coping would improve, previous study found that 7- to 17-year-olds with FAP’s responses fell closer to the middle of the catastrophizing subscale of the Pain Response Inventory, which includes items such as “When you have a bad stomach ache, how often do you think to yourself that it’s never going to stop?” [27,30]. Taken together, these studies’ findings suggest that younger children may be more optimistic than older children about the stability of their pain. Children’s responses regarding the global impact of Teddy’s pain (e.g., whether his pain caused problems at home or school or kept him from having fun), were more variable. These results are consistent with previous study findings that 8- to 18-year-old pediatric patients with FAP showed varying levels of functional disability that corresponded to their coping styles: self-reliant copers, who use acceptance, minimization, and self-encouragement to cope with pain, tended to have low levels of functional disability; avoidant copers, who avoid social contact and keep their pain experience private, had the highest functional disability of any coping profile [26]. Overall, 80% of children in our sample were able to generate at least one strategy Teddy could use to make his pain better, including strategies related to health evaluation, changes in nutrition, increased hydration, body-focused strategies, mind-focused strategies, activity engagement, and socially-oriented strategies. Given the previous study’s findings associating functional disability with avoidant coping profiles [26], it may be important to capitalize on young children’s tendency to generate active coping strategies before these avoidant coping profiles emerge.

While most children in this sample endorsed optimistic pain beliefs, a subset of children were pessimistic about Teddy’s pain. Approximately 10.5% of children did not think that Teddy would get better at dealing with pain, and 5.3% thought he would have the pain forever. One-fifth of children did not generate any strategies for making Teddy’s pain better. Among this group, only one child (2.6% of the study sample) believed both that Teddy’s pain would last forever and that Teddy would not get better at dealing with the pain; however, this child did generate multiple pain coping strategies. One child (2.6% of sample) believed that Teddy would not get better at dealing with the pain and did not generate any coping strategies, but they indicated that Teddy would not have the pain forever. The finding that none of the children endorsed entirely negative or helpless beliefs highlights the importance of early intervention while children have flexibility in their beliefs about pain.

Children indicated that Teddy’s pain interfered with many types of activities, including health behaviors, school, and recreational activities. Additionally, some coping strategies were developmentally appropriate but could lead to excessive help seeking if not intervened upon early. 15.8% of children suggested that Teddy go to the doctor or get a vaccination. A medical visit is appropriate for pain at times; however, this finding may be an early indicator of overutilization of the health care system. Previous studies have linked childhood functional gastrointestinal disorders to substantial financial burden to families and health care systems [46,47,48]. As a function of both child and parent decisions, health care seeking in children is complicated. While overutilization may be a maladaptive pattern for individuals with functional gastrointestinal disorders, the decision about when to visit a health care provider is nuanced by multiple factors, including children’s reports of pain intensity, their sense of threat about their pain, and the caregiver’s influence over the decision. This complex issue highlights the need to help children and parents to develop mastery related to children’s symptoms and guidance about when seeking health care is optimal.

In regard to the relation between pain intensity and beliefs, children whose parents perceived their morning pain intensity as higher, on average, were more likely to believe that Teddy’s pain caused problems at school; however, child ratings of pain in the morning did not show the same relation with this belief. Surprisingly, children whose parents rated their pain as reaching higher levels of intensity over the course of the day were more likely to believe that Teddy’s pain would not last forever. However, most correlations between child- and parent-rated pain intensity and frequency and child pain beliefs were not significant. While there was correspondence between a child’s pain beliefs and a parent’s rating of pain intensity in certain circumstances, findings were inconsistent and puzzling. This incongruence between child and parent pain reports and beliefs highlights the need to better understand what children and parents are noticing and responding to when asked about pain and develop new measures and strategies to address the complexity and nuance of multi-informant ratings of internal experiences.

In Salkovskis’s cognitive-behavioral therapy model of adult health anxiety, the two processes that are involved in maintaining patients distorted beliefs are first, misinterpreting body symptoms as indicating a serious or threatening health problem and second, using ‘safety-seeking behavior’ to cope with this anxiety, behaviors that may have the inadvertent consequence of maintaining pain. In this study, parent ratings of child pain and the child’s pain beliefs were correlated. While direction of causality cannot be determined, several interpretations are possible. One hypothesis is that a parent’s perception of their child’s pain plays a role in how the child interprets the severity of their symptoms. Safety behaviors (e.g., palpating the abdomen regularly, body vigilance, limiting the diet, over-resting) can prevent a child from learning that they can cope with a situation because they believe they managed it only *because* of the safety behavior. Our small study demonstrates that even at a young age, children generate numerous coping strategies for pain management. Further research is needed to elucidate the conditions under which creative coping strategies morph into the maladaptive safety behaviors of health anxiety. An alternative hypothesis is that parents are accurately reflecting the experiences communicated by their child. Thus, teaching young children how to distinguish the meanings and severity of different bodily signals, teaching and showing children how to respond to the communicative messages of bodily signals adaptively, and encouraging active approach behaviors may be helpful strategies.

In regard to age-related differences in pain beliefs, the older the children were, the more likely they were to believe that Teddy would be feeling better when he was a year older. Older children also believed that Teddy could do a variety of activities with a higher intensity of pain than younger children indicated, on average. One possible explanation for these findings is that older children have had more opportunities to try engaging in activities when they have pain, proving to themselves that it is possible. Another is that older children may have experienced some improvements or fluctuations in pain, learning that it can and does change. An additional possibility is that older children may be more sensitive to the social influences of a situation and thereby more likely to provide responses they think the researcher expects.

In regard to impairment, children indicated that Teddy could tolerate significantly more pain when watching television or eating a healthy food than when going to school. The levels of pain that they rated Teddy could tolerate when doing other activities (e.g., playing a favorite sport, playing with a friend, eating a favorite food) were not significantly different than the level of pain he could tolerate when going to school in the sample as a whole. This finding adds complexity to previous studies which have associated childhood FAP with school avoidance [49,50]. Historically, childhood FAP has been mistakenly viewed as a diagnosis of exclusion, and as a result, the pain in children with FAP is viewed as a complex combination of biological and potential psychological factors. However, our findings show that the children believed Teddy’s pain threshold to be approximately the same for school and other activities, including playing with a friend, playing a sport, and eating a favorite food. Thus, these results from the Teddy Bear Paradigm indicate that children with FAP, even at a young age, are unlikely to be using pain as an excuse to avoid school even though school avoidance has previously been cited as a consequence of functional gastrointestinal disorders [50,51]. It may be that school avoidance occurs as children age, yet another support for early intervention for pain.

One limitation of these analyses related to pain tolerance is that school is an enjoyable activity for many children; therefore, it may not have been an appropriate reference group for these comparisons. Additionally, in an exploratory analysis of age differences in ratings of pain tolerance, results remained significant for 7- to 9-year-olds but not 5- to 6-year-olds. This limitation could be due to the study being underpowered to detect differences in younger children, or that 5- to 6-year-olds were too young to understand the question. While there was some variability in younger children’s responses, future research is needed to investigate whether younger children are able to differentiate pain tolerance thresholds for different activities. Nonetheless, if it is true that the child’s own pain tolerance can be inferred from their description of Teddy’s, these findings show that young children can calibrate their pain tolerance depending on the context. They believe that their pain will be easier to tolerate if they rest, or engage in healthy eating, and this may be the implicit message that they receive from parents, school, and/or society. On one hand, engaging in healthy eating can be a positive healthy behavior. On the other hand, these beliefs can become problematic in certain chronic conditions. For example, findings from the literature on pediatric chronic pain describe that rather than alleviating pain, too much rest can exacerbate chronic pain [52]: avoiding activities entirely weakens the body and therefore worsens pain. Additionally, changing eating patterns in order to avoid aversive internal sensations may be a vulnerable learning event for the emergence of disordered eating [53,54].

The Teddy Bear Interview may offer advantages in use with children with FAP. By externalizing their pain beliefs, children may have been able to approximate their own explanatory styles related to pain. This externalizing process also offers children a context to creatively think about pain and coping strategies. It may even possess therapeutic potential in allowing children to think about pain through a different lens and potentially generate self-compassion by imagining someone else experiencing and coping with pain that is similar to their own.

Overall, these results suggest that early intervention may be important for children with FAP. In general, most children were optimistic about pain improvement and already resourceful in generating strategies for coping with pain. Therapy can provide a context to facilitate children and parents to enact helpful strategies while preventing the emergence of maladaptive safety-seeking behaviors. Strategies that teach children how to distinguish between the interpretations of different bodily sensations, thereby improving not only their self-awareness, but also the precision of their communication, may help facilitate parents’ adaptive responses to child pain. In turn, helping parents to facilitate adaptive responses to pain may be important in shaping that child’s ongoing pain beliefs. For example, as previous studies have found a link between chronic pain, school anxiety, and avoidance, early intervention may be especially important to allow children to undergo corrective experiences about how much pain they can endure and still attend activities such as school [55,56,57,58]. Finally, the presence of a significant percentage of children that could not generate any strategies to manage pain further emphasizes the importance of early intervention.

Limitations of this exploratory analysis include its small sample size. Future studies should seek to evaluate pain beliefs in a larger sample of young children with FAP in order to verify these results. Additionally, while the Teddy Bear Interview possesses a high level of face validity and is based on the previously tested Teddy Bear Hospital paradigm, it has not been tested in a large sample of children with FAP. Another constraint of these findings is that because children were answering questions about Teddy’s pain, rather than their own, it is possible that their beliefs about their own pain may differ from these findings. Importantly, this sample was limited in its representation of the overall population of children with FAP demographically. Previous studies have found no difference in prevalence of FAP among different races and ethnicities [59,60]; therefore, future studies should seek to characterize beliefs in a more diverse sample.

In summary, the majority of young children with FAP in this study were optimistic about the prognosis for Teddy’s pain. Most children generated a variety of strategies for coping with pain, and interventions need to optimize creative and flexible problem solving coupled with curious body awareness, while avoiding the development of rigid safety behaviors. How children decipher body sensations, their emotional reactions to these sensations, and how parents perceive their child’s pain communications may be important in the development of children’s ongoing beliefs regarding the strengths and wisdom of their bodies, their capacities for pain tolerance, and the stability of their pain experiences. All of this highlights the critical role of early intervention when children are first learning to decipher the meanings of body sensations, with the aim to prevent future pain, impairment, and persistent mental health conditions.

## Figures and Tables

**Table 1:** Baseline parent proxy PROMIS anxiety and depressive symptoms scores for Teddy bear lab sample.

Symptoms	Severity Level				Sample Average
	Within Normal Limits	Mild	Moderate	Severe	
Anxiety	26.3% (10)	15.8% (6)	55.3% (21)	2.6% (1)	M = 9.50 SD = 4.68
Depression	44.7% (17)	21.1% (8)	31.6% (12)	2.6% (1)	M = 4.53 SD = 3.62

Note. Anxiety and depressive symptoms were measured using the Patient Reported Outcomes Measurement Information System (PROMIS) Parent Proxy Short Form v1.0–Anxiety 8a and PROMIS Parent Proxy Short Form v1.0–Depressive Symptoms 6a. Possible raw scores for the anxiety measure range from 0 to 32, and depressive symptoms from 0 to 24. Raw scores from these measures are associated with T-scores, with T-scores up to 50 associated with symptoms within normal limits, 50 to 55 with mild symptoms, 55 to 65 with moderate, and 65 and over with severe.

**Table 2:** Demographic information of study sample relative to the full sample.

Demographic	Duke Tummy Study (*n* = 107)	Teddy Bear Lab (*n* = 38)
Gender	Male: 43.9% (47)	Male: 39.5% (15)
	Female: 56.1% (60)	Female: 60.5% (23)
Age	M = 7.5 (SD = 1.4)	M = 7.2 (SD = 1.2)
	Aged 5–6: 36.4% (39)	Aged 5–6: 39.5% (15)
	Aged 7–9: 63.6% (68)	Aged 7–9: 60.5% (23)
White	76.6% (82)	84.2% (32)
Black	12.2% (13)	13.2% (5)
Asian	1.9% (2)	0% (0)
Mixed	8.4% (9)	2.6% (1)
Unknown Race	0.9% (1)	0% (0)
Hispanic	4.7% (5)	2.6% (1)

Note. The sample for the Teddy Bear Lab included all participants in enrolled in a clinical trial for functional abdominal pain from the time the interview was implemented until the end of the trial. There were no differences in age, sex, race or ethnicity of this subsample. The mean age of participants that completed the Teddy interview (*M* = 7.2, SD = 1.2) did not significantly differ from the full sample (*M* = 7.5, SD = 1.4), *t*(37) = −1.34, *p* = 0.188. Race distribution of the Teddy sample did not significantly differ from the distribution of the full sample, *χ*^2^(2) = 1.75, *p* = 0.417. Ethnicity distribution of the Teddy sample did not significantly differ from the distribution of the full sample, *χ*^2^(1) = 0.37, *p* = 0.541. Sex distribution of the Teddy sample did not significantly differ from the distribution of the full sample, *χ*^2^(1) = 1.33, *p* = 0.249.

**Table 3:** Correlations among Children’s Pain Frequency, Intensity, and Beliefs.

Variable	1	2	3	4	5	6	7	8	9	10	11	12	13	14
1. Pain frequency	—													
2. Morning pain intensity, parent rated	0.46 **	—												
3. Morning pain intensity, child rated	0.38 *	0.80 **	—											
4. Before dinner pain intensity, parent rated	0.28	0.57 **	0.61 **	—										
5. Before dinner pain intensity, child rated	0.30	0.51 **	0.76 **	0.74 **	—									
6. Highest level of pain intensity, parent rated	0.43 **	0.59 **	0.55 **	0.48 **	0.51 **	—								
7. How strong or weak do you think Teddy is, compared to other Teddys?	−0.07	−0.04	−0.22	−0.12	−0.18	−0.06	—							
8. Do you think Teddy will have the pain forever?	−0.02	0.10	0.11	0.29	0.21	0.36 *	0.02	—						
9. Do you think the pain will bother Teddy forever?	−0.23	0.11	0.03	−0.06	−0.02	−0.06	0.05	0.25	—					
10. Do you think Teddy will get better at dealing with the pain?	−0.03	0.08	0.05	0.18	0.13	−0.13	−0.03	−0.20	0.27	—				
11. When Teddy is a year older, how do you think Teddy will be feeling?	0.16	0.13	0.15	−0.16	−0.11	−0.11	0.10	−0.38 *	−0.03	0.11	—			
12. Does Teddy’s tummy pain cause problems at home?	−0.01	−0.12	0.02	0.05	0.22	0.01	0.10	0.13	0.07	0.21	0.03	—		
13. Does Teddy’s tummy pain cause problems at school?	−0.11	−0.32^*^	−0.21	−0.03	0.00	−0.21	0.08	−0.20	−0.13	−0.05	−0.09	0.57 **	—	
14. Does Teddy’s tummy pain stop him from having fun most of the time?	0.12	−0.21	−0.17	−0.28	−0.13	−0.15	−0.04	−0.02	−0.11	−0.22	−0.03	0.14	0.00	—

Note. *n* = 38. * *p* < 0.05. ** *p* < 0.01.

## Data Availability

De-identified data will be made available upon request.

## Appendix A

Teddy Bear Interview

You are now going to be the doctor. Teddy is your patient. Teddy has pain like you have.

**General Pain Information**

1. Can you point to Teddy’s pain?

Is the pain anywhere else?

2. How is Teddy feeling when he’s in pain?

3. Can you think of ways to make Teddy’s pain better?

________________________________________________________________________

**Personal**

4. Teddy has pain just like you. Point to the Teddy that shows how strong or weak you think Teddy is, compared to other Teddys.

**Stable**

5. Do you think Teddy will have the pain forever?

Yes Maybe No

6. Do you think the pain will bother Teddy forever?

Yes Maybe No

7. Do you think Teddy will get better at dealing with the pain?

Yes Maybe No

**Global**

8. When Teddy is a year older, how do you think Teddy will be feeling?

Better The same Worse

9. Does Teddy’s tummy pain cause problems at home?

Yes Maybe No

10. Does Teddy’s tummy pain cause problems at school?

Yes Maybe No

11. Does Teddy’s tummy pain stop him from having fun most of the time?

Yes Maybe No

**School**

12. What kinds of things can Teddy still do, even when he is in pain? Do you think Teddy can still go to school with Tummy pain?

Yes Maybe No

13. What is the most pain that Teddy can have and still go to school?

14. When Teddy goes to school with tummy pain, do you think he is weak, a little weak, in the middle, a little strong, or strong?

**Sport**

15. Do you think Teddy can still play a favorite sport with tummy pain?

Yes Maybe No

16. What is the most pain that Teddy can have and still play a favorite sport?

17. When Teddy plays a favorite sport with tummy pain, do you think he is weak, a little weak, in the middle, a little strong, or strong?

**Play with Friends**

18. Do you think Teddy can still play with a friend with tummy pain?

Yes Maybe No

19. What is the most pain that Teddy can have and still play with friends?

20. When Teddy plays with his friends when he has tummy pain, do you think he is weak, a little weak, in the middle, a little strong, or strong?

**Watch TV**

21. Do you think Teddy can still watch TV with tummy pain?

Yes Maybe No

22. What is the most pain that Teddy can have and still watch TV?

23. When Teddy watches TV when he has tummy pain, do you think he is weak, a little weak, in the middle, a little strong, or strong?

**Read a Book**

24. Do you think Teddy can still read a book with tummy pain?

Yes Maybe No

25. What is the most pain that Teddy can have and still read a book?

26. When Teddy reads a book when he has tummy pain, do you think he is weak, a little weak, in the middle, a little strong, or strong?

**Eat a Favorite Food**

27. Do you think Teddy can still eat a favorite food with tummy pain?

Yes Maybe No

28. What is the most pain that Teddy can have and still eat a favorite food?

29. When Teddy eats his favorite food with tummy pain, do you think he is weak, a little weak, in the middle, a little strong, or strong?

**Eat a Healthy Food**

30. Do you think Teddy can still eat a healthy food with tummy pain?

Yes Maybe No

31. What is the most pain that Teddy can have and still eat a healthy food?

32. When Teddy eats a healthy food with tummy pain, do you think he is weak, a little weak, in the middle, a little strong, or strong?

**Other Ideas**

33. Can you think of another activity that Teddy’s pain gets in the way of?

____________________________________________________________________

34. Do you think Teddy can still do this activity with tummy pain?

Yes Maybe No

35. What is the most pain that Teddy can have and still do this activity?

36. When Teddy does this activity with tummy pain, do you think he is weak, a little weak, in the middle, a little strong, or strong?

37. Is there anything else that is important for us to know about Teddy’s pain?

_________________________________________________________________
